# Metabolic profiles derived from residual blood spot samples: A longitudinal analysis

**DOI:** 10.12688/gatesopenres.12822.1

**Published:** 2018-05-30

**Authors:** Malia S.Q. Murphy, Steven Hawken, Wei Cheng, Lindsay A. Wilson, Monica Lamoureux, Matthew Henderson, Beth Potter, Julian Little, Pranesh Chakraborty, Kumanan Wilson

**Affiliations:** 1 Clinical Epidemiology Program, Ottawa Hospital Research Institute, Ottawa, ON, K1Y 4E9, Canada; 2 Newborn Screening Ontario, Children’s Hospital of Eastern Ontario, Ottawa, ON, K1H 5B2, Canada; 3 School of Epidemiology and Public Health, University of Ottawa, Ottawa, ON, K1G 5Z3, Canada

**Keywords:** Newborn screening, policy, sample stability, dried blood spots, storage conditions

## Abstract

**Background: **Secondary use of newborn screening dried blood spot samples include use for biomedical or epidemiological research. However, the effects of storage conditions on archival samples requires further examination. The objective of this study was to determine the utility of residual newborn samples for deriving reliable metabolic gestational age estimates.

**Methods: **Residual newborn dried blood spot samples that had been stored for 2-, 4-, 6-, or 12-months in temperature controlled (21°C) conditions were re-analyzed for the full panel of newborn screening analytes offered by a provincial newborn screening lab in Ottawa, Canada. Data from re-analyzed samples were compared to corresponding baseline newborn screening values for absolute agreement, and Pearson and intraclass correlation. Performance of a gestational age estimation algorithm originally developed from baseline newborn screening values was then validated on data derived from stored samples.

**Results: **A total of 307 samples were used for this study. 17-hydroxyprogesterone and newborn hemoglobin profiles measured by immunoassay and high-performance liquid chromatography, respectively, were among the most stable markers across all time points of analysis. Acylcarnitines exhibited the greatest degree of variation in stability upon repeat measurement. The largest shifts in newborn analyte profiles and the poorest performance of metabolic gestational age algorithms were observed when samples were analyzed 12-months after sample collection.

**Conclusions: **Duration of sample storage, independent of temperature and humidity, affects newborn screening profiles and gestational age estimates derived from metabolic gestational dating algorithms. When considering use of dried blood spot samples either for clinical or research purposes, care should be taken when interpreting data stemming from secondary use.

## Introduction

Newborn screening is a public health initiative that tests infants shortly after birth for rare but treatable diseases. The screening process entails the collection of 4–5 drops of newborn blood by heel prick, typically within 24–72 hours of life. Newborn samples are collected onto filter paper and sent to accredited laboratories where the dried blood spots are used to screen infants for risk of developing diseases included in the laboratory’s screening panel. Although newborn screening is typically completed within the first few days of birth, secondary use of samples is not uncommon. Beyond provision of health care, residual dried blood spot samples may be used for quality assurance to improve existing tests and programs, used under legal warrant or court order, or used for biomedical or epidemiological research ^1^,^2^.

Emerging secondary uses of newborn screening data include using screening profiles for biological modelling. For example, gestational age estimation algorithms based on a combination of newborn screening analytes and clinical covariates such as sex and birthweight have emerged as novel alternatives for accurately categorizing infants across preterm birth categories. Postnatal gestational age dating based on newborn metabolic profiles generated from dried blood spot samples provides the opportunity to establish preterm birth estimates ^3^,^5^ for jurisdictions for which data on preterm birth are currently lacking or inaccurate due to bias in population sampling and non-standardized use of clinical preterm birth thresholds ^6^. Given the breadth of possible secondary uses of newborn screening samples, it is important to understand the effect of storage conditions on newborn screening samples. 

In this study, we sought to determine longitudinal changes in metabolic profiles derived from residual blood spot samples from a provincial newborn screening facility in Ottawa, Canada. The effects of longitudinal changes in metabolic profiles on the performance of gestational age estimation models as a result of storage were determined.

## Methods

### Newborn Screening Ontario

The data for this study were derived from a quality assurance project run through Newborn Screening Ontario (NSO), located at the Children’s Hospital of Eastern Ontario. NSO is the provincial program that coordinates newborn screening in Ontario, Canada, screening more than 145,000 infants each year for over 90 analytes and analyte ratios.

After testing at NSO, newborn dried blood spot samples from healthy infants are temporarily stored on-site at 21°C, after which they are sent to a secure off-site facility as part of the newborn medical record. These stored samples can be used for secondary purposes, including use for method development, method comparisons and transfer of screening thresholds.

### Sample collection and analysis

Archival screen-negative dried blood spot samples collected over the course of 2016-2017, that had been stored for 2-, 4-, 6-, or 12-months after initial analysis were used for this study. As per standard newborn screening policy, initial analysis of all samples occurred within two weeks of collection. The sample set was enriched to include approximately 40–50% preterm infants by random selection of available samples from infants born ≥ or <37 weeks gestation. Eight 3.2 mm diameter circular samples were punched from each dried blood spot sample for first tier testing of each of the following analytes: hemoglobin profiles; 17α hydroxyprogesterone (17-OHP); thyroid stimulating hormone (TSH); a panel of 12 amino acids and 31 acylcarnitines; t-cell receptor excision circles (TREC); biotinidase activity; and galactose-1-phosphate uridylyltransferase activity. Hemoglobin profiles were determined by high performance liquid chromatography on a Bio Rad Variant nbs system; neonatal 17-OHP, and TSH were measured using a PerkinElmer AutoDELFIA® Immunoassays; amino acid and acylcarnitine analysis was performed by electrospray ionization tandem mass spectrometry (Waters TQ Detector); total TREC copy number was measured by quantitative polymerase chain reaction using a ThermoFisher Scientific Viia 7; biotinidase and galactose-1-phosphate uridyltransferase levels were measured using the Astoria-Pacific SPOTCHECK® Pro system. For each sample included in the study, analyses conducted at each storage time point were compared with the original baseline analyses for the same newborn.

### Statistical analysis

***Agreement between paired baseline and stored metabolic profiles.*** Descriptive statistics were generated for the cohort. All analyte and clinical variables were standardized to a larger Ontario reference cohort by subtracting the mean and dividing by the standard deviation of the reference cohort ^7^. For each storage time point Pearson and intraclass correlation ^8^ coefficients were calculated between paired baseline and stored sample analyte levels. Two-sided Wilcoxon paired tests were used to compare baseline and storage data. Boxplots were used to describe changes in each analyte from baseline to paired storage time point in standard deviation units.

***Validation of metabolic gestational age estimation models.*** Our group has previously developed and validated gestational age estimation algorithms derived from newborn screening profiles and other clinical covariates ^3^,^7^,^9^. Linear regression models were developed to estimate continuous gestational age, and logistic models were fit to classify infants as term (≥ 37 completed gestational age weeks) or preterm (<37 completed gestational age weeks). Published gestational age estimation models were developed and validated using metabolic profiles generated within the standard newborn screening timeframe ^3^,^7^,^9^.

To determine the impact of delayed analysis and storage on the performance of gestational age estimation models, we externally validated the performance of our models in samples analyzed at baseline (time 0) and after 2-, 4-, 6- and 12- months of storage. Samples where secondary screening could not be completed due to insufficient sample volume were excluded from model testing. Model coefficient estimates from our previously published models ^7^ were fixed and used to score each infant’s metabolic profile to generate an estimated gestational age. Root mean square error (RMSE) was used to evaluate model performance. The mean square error (MSE) was calculated as the average of the squared differences of each estimated gestational age compared to each actual (ultrasound-validated) gestational age. The RMSE, the square root of MSE, in units of gestational age in weeks, provides an intuitive measure of goodness of fit of the model. For logistic models, we measured area under the receiver-operator characteristic curve (AUC). The performance of each gestational age estimation model was validated as previously published ^7^:

- Model 1: containing only the clinical factors of infant sex, birthweight, and multiple birth (yes,no)
- Model 2: Model 1 + newborn screening analytes and analyte ratios including acylcarnitines, amino acids and enzyme markers.

All analyses were conducted using SAS software version 9.4 ^10^, and R version 3.32 ^11^.

## Results

### Sample characteristics

A total of 307 samples were analysed for this study. 74 samples were procured 2 months after initial analysis; 77 at 4 months; 78 at 6 months; and 78 at 12 months. The majority (68.1%) of samples were obtained from infants with a birthweight of ≥2500g, and 52.8% of samples were from term infants (born ≥37 weeks gestational age). Newborn samples were collected earlier among term infants (63.3±117.1 hrs after birth) than preterm infants (81.4±142.6 hrs after birth). A summary of newborn characteristics is provided in Table 1.

**Table 1.** Summary of patient characteristics.

	All samples n=307	Duration of sample storage			
		2-months n=74	4-months n=77	6-months n=78	12-months n=78
Sex					
Male, n(%)	144 (46.9)	34 (45.9)	40 (51.9)	37 (47.4)	33 (42.3)
Female, n(%)	162 (52.8)	40 (54.1)	37 (48.1)	40 (58.3)	45 (57.7)
Unknown, n(%)	1 (0.3)	0 (0.0)	0 (0.0)	1 (1.3)	0 (0.0)
Birthweight, g	2846.0±858.3	2927.0±885.7	2840.6±939.2	2794.9±817.5	2825.5±798.6
≥4,000g, n(%)	23 (7.5)	5 (6.8)	8 (10.4)	3 (3.8)	7 (9.0)
2500g to <4000g, n(%)	186 (60.6)	51 (68.9)	41 (53.2)	49 (62.8)	45 (57.7)
1500g to <2500g, n(%)	69 (22.5)	11 (14.9)	18 (23.4)	19 (24.4)	21 (26.9)
1000g to <1500g, n(%)	14 (4.6)	4 (5.4)	4 (5.2)	3 (3.8)	3 (3.8)
<1000g, n(%)	10 (3.3)	2 (2.7)	4 (5.2)	3 (3.8)	1 (1.3)
Unknown	5 (1.6)	1 (1.4)	2 (2.6)	1 (1.3)	1 (1.3)
Gestational age, wks	36.9±3.5	37.1±4.0	36.7±3.6	36.8±3.6	37.1±2.8
≥37 weeks, n(%)	162 (52.8)	45 (60.8)	39 (50.6)	39 (50.0)	39 (50.0)
<37 weeks, n(%)	145 (47.2)	29 (39.2)	38 (49.4)	39 (50.0)	39 (50.0)
Multiple birth, n(%)	36 (11.7)	11 (14.9)	7 (9.1)	8 (10.3)	10 (12.8)
Newborn age at sample collection, hrs	75.5±134.2	96.3±154.1	80.2±143.3	86.2±155.2	40.4±54.2
Term infants	40.0±64.1	34.2±17.4	29.2±5.9	63.2±127.3	34.5±10.1
Preterm infants	115.1±175.2	192.6±213.6	132.5±191.2	109.3±177.5	46.3±76.0

Data are presented as mean±SD unless otherwise specified.

### Changes in metabolic data as a result of storage

**Figure 1.**
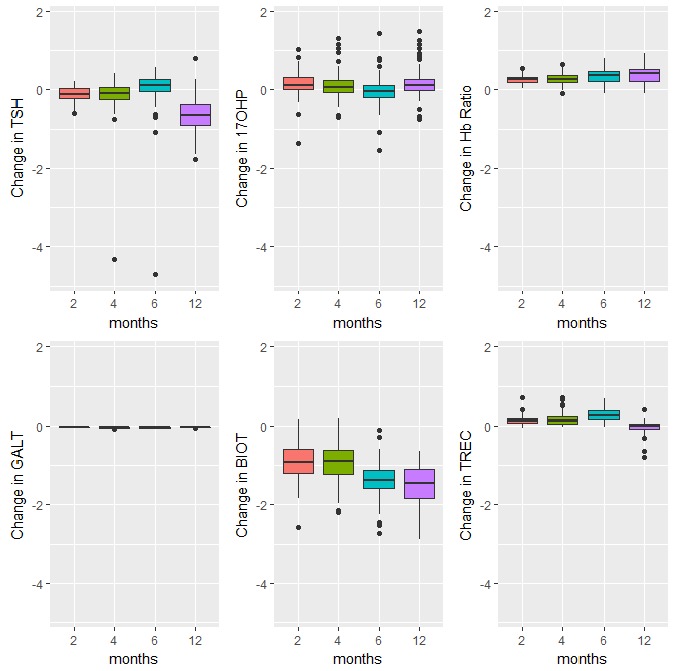
Endocrine, enzyme and other markers. Boxplots of the changes in analyte levels after 2-, 4-, 6-, and 12-months of storage from baseline. The most variable marker in this category was biotinidase (BIOT). The lower whisker = smallest observation greater than or equal to lower hinge - 1.5 * IQR, and the upper whisker = largest observation less than or equal to upper hinge + 1.5 * IQR.

Box plots depicting changes in standardized analyte concentration determined within one week of sample collection and after storage are provided in Figure 1– Figure 3.

**Figure 2.**
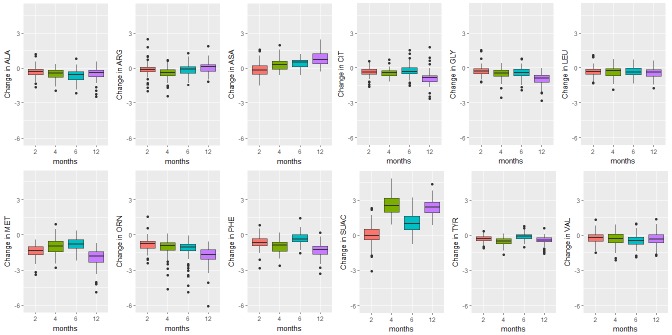
Amino acids. Boxplots of the change in analyte levels after 2-, 4-, 6-, and 12-months of storage from baseline. The most variable marker in this category was argininosuccinic acid (SUAC). The lower whisker = smallest observation greater than or equal to lower hinge - 1.5 * IQR, and the upper whisker = largest observation less than or equal to upper hinge + 1.5 * IQR.

**Figure 3.**
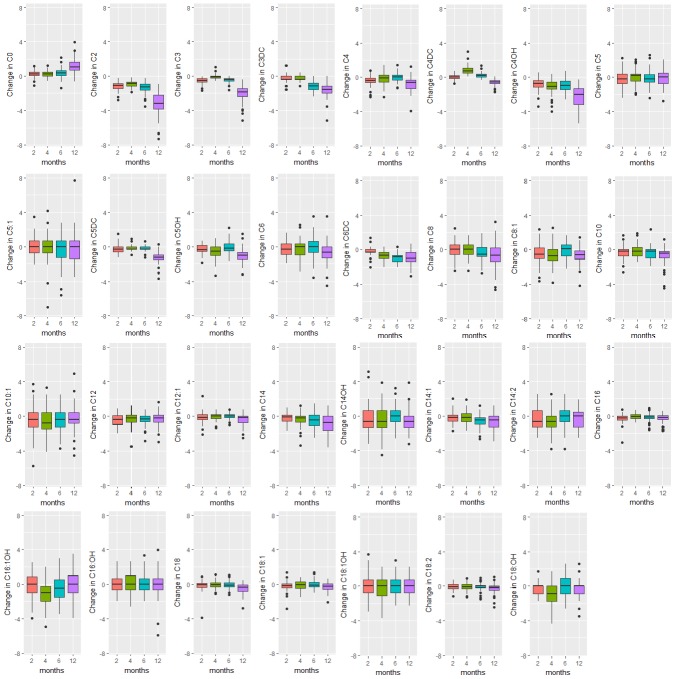
Acylcarnitines. Boxplots of the change in analyte levels after 2-, 4-, 6-, and 12-months of storage from baseline. The lower whisker = smallest observation greater than or equal to lower hinge - 1.5 * IQR, and the upper whisker = largest observation less than or equal to upper hinge + 1.5 * IQR.

The majority of analyte levels (30 out of 48) were consistent at 2-months when compared to baseline levels (Pearson r≥0.8). 25 out of 48 of the measured analytes were highly correlated with baseline levels both 2-months and 4-months after collection (Pearson r≥0.8). Analytes exhibiting rapid degradation between initial analysis and 2 months after collection (Pearson r<0.5) were the amino acid argininosuccinic acid, and acylcarnitines C10:1, C14:OH, C16OH, C18:1OH, C18OH, C5:1. The endocrine hormone 17-hydroxyprogesterone (17-OHP) and relative levels of fetal hemoglobin peaks, taken by the ratio (HbF+F1)/(HbF+F1+A) were consistently the top two correlated analytes across all time points of analysis. Pearson and Intra-class correlations (ICC) with 95% CIs comparing baseline values to each time point of analysis, and Wilcoxon test results are provided in Supplementary File 1.

### Validation of gestational age algorithms

The performance of the linear regression models in providing continuous estimates of gestational age and correctly identifying gestational age within 1 and 2 weeks of ultrasound validated gestational age are summarized in Table 2 and Table 3, respectively. Application of linear models to fresh baseline samples revealed that a model including metabolic parameters (Model 2) consistently provided better estimates of gestational age than a clinical model limited to birthweight, sex and multiple birth status (Model 1). Metabolite models outperformed clinical estimates when metabolite data were derived from samples that had been stored for 2 months, 4 months and 12 months.

**Table 2.** Performance of models to provide continuous estimates of gestational age.

	2 months storage (n=60)			4 months storage (n=61)			6 months storage (n=65)			12 months storage (n=38)		
	Fresh	Stored	∆	Fresh	Stored	∆	Fresh	Stored	∆	Fresh	Stored	∆
Model 1	1.42 wks		-	1.35 wks		-	1.11 wks		-	1.60 wks		-
Model 2	1.21 wks	1.20 wks	-0.00	1.04 wks	1.08 wks	+0.04	0.93 wks	1.16 wks	+0.24	1.39 wks	1.48 wks	+0.10
∆	-0.22	-0.22		-0.31	-0.27		-0.19	+0.05		-0.22	-0.12	

Data are expressed as RMSE, root mean squared error (average absolute deviation of ultrasound-validated vs. model estimated gestational in weeks); **∆**, Black=unchanged, Green=improvement in model accuracy, Red=attenuation in model accuracy

**Table 3.** Proportion of samples with gestational age correctly estimated within 1 week, 2 weeks of ultrasound-validated gestational age.

	2 months storage (n=60)			4 months storage (n=61)			6 months storage (n=65)			12 months storage (n=38)		
	Fresh	Stored	∆	Fresh	Stored	∆	Fresh	Stored	∆	Fresh	Stored	∆
Model 1	45.0%, 88.3%		-	54.1%, 83.6%		-	64.6%, 92.3%		-	47.4%, 73.7%		-
Model 2	63.3% 88.3%	65.0% 95.0%	+1.7%, +6.7%	70.5%, 95.1%	63.9% 93.4%	-6.6%, -1.6%	76.9%, 95.4%	55.4%, 92.3%	-21.5%, -3.1%	52.6%, 89.5%	60.5% 81.6%	+7.9%, -7.9%
∆	+18.3%, 0.0%	+20.0%, +6.7%		+16.4%, +11.5%	+9.8%, +9.8%		+12.3%, +3.1%	-9.2%, 0.0%		+5.3%, +15.8%	+13.2%, +7.9%	

Data are expressed as percentage classified within 1 week, percentage classified within 2 weeks; **∆**, Black=unchanged, Green=improvement in model accuracy, Red=attenuation in model accuracy

Whereas the performance of metabolite models was similar for data derived after 2 and 4 months of storage compared to paired baseline values (each within 0.04 weeks RMSE and 7% of the proportion of infants correctly classified within 1 and 2 weeks of ultrasound validated gestational age), results after 6 months and 12 months of storage were more variable. Metabolite data measured after 6 months of sample storage yielded gestational age estimates that were 0.24 weeks less accurate than estimates derived from fresh samples. Here, gestational age was correctly identified within 1 week for 21.5% fewer infants, and within 2 weeks for 3.1% fewer infants. After 12 months of storage, estimates were 0.1 week less accurate, and gestational age was correctly identified within 1 week for 7.9% more infants, but within 2 weeks for 7.9% fewer infants.

**Figure 4.**
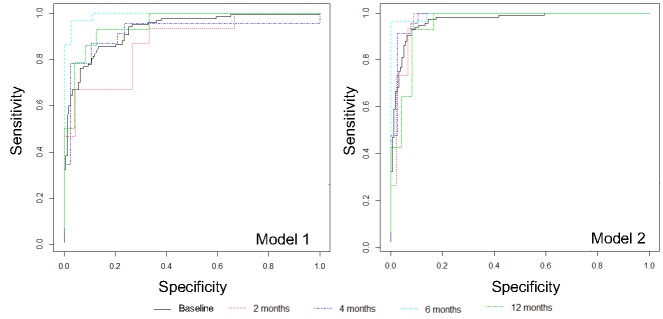
Performance of models to determine gestational age across dichotomous categories of preterm birth by time point of sample analysis. Metabolic models consistently provide more accurate estimates of gestational age, regardless of age of sample at the time of analysis, AUC all >0.95. Model 1, clinical variables only; Model 2, clinical variables + metabolite markers.

We also evaluated the capacity of published models to accurately categorize samples across dichotomous gestational age categories (term, ≥37 weeks gestational age; preterm, <37 weeks gestational age) by logistic regression ( Figure 4). As with the linear regression models, Model 2 consistently provided more accurate estimation of gestational age at baseline (AUC0.968 [95%CI 0.945, 0.991]) and after 2-months (AUC 0.970 [0.909, 1.00]), 4-months (AUC 0.981 [0.940,1.000]), 6-months (AUC 0.995 [0.977, 1.000]) and 12-months (AUC 0.955 [0.876, 1.000]) of storage compared to estimates derived from Model 1. The incremental improvement in gestational age estimation from Model 1 to Model 2 was attenuated when samples had been stored for 6- and 12-months compared to when analyzed at baseline and after 2- or 4- months of storage. Logistic regression model performance metrics are provided in Supplementary File 1.

A comparison of ultrasound-validated preterm birth rates and algorithm-based estimates are provided in Table 4. In the overall cohort, 42.6% of infants were born preterm. The metabolic gestational age model, Model 2, provided better estimates of preterm birth compared to the clinical model when applied to baseline metabolic data (40.4% vs 34.9%), and also when applied to data obtained from stored samples. Misclassification was greatest when the metabolic model was applied to data captured after samples had been stored for 12-months (7.9% higher than ultrasound-validated preterm birth rate).

**Table 4.** Estimated proportions of pre-term infants using metabolic-based algorithm.

	Baseline n=275		2 month storage n=60		4 month storage n=61		6 month storage n=65		12 month storage n=38	
**Ultrasound-** **validated** **proportions** **of preterm** **infants**	117 (42.6%)		15 (25.0%)		23 (37.7%)		29 (44.6%)		14 (36.8%)	
**Algorithmic** **estimates**	Model 1	Model 2	Model 1	Model 2	Model 1	Model 2	Model 1	Model 2	Model 1	Model 2
	96 (34.9%)	111 (40.4%)	11 (18.3%)	15 (25.0%)	17 (27.9%)	21 (34.4%)	24 (36.9%)	29 (44.6%)	19 (50.0%)	17 (44.7%)
**∆**	↓7.7%	↓2.2%	↓6.7%	0.0%	↓9.8%	↓3.3%	↓7.7%	0.0%	↑13.2%	↑7.9%

Data are presented as n (%). Model 1, clinical variables only; Model 2, clinical variables + metabolite markers.

## Discussion

In this study we provide insight into the stability of residual newborn screening samples, and the impact of year-long storage on screening profiles. Hemoglobin profiles, amino acids, and endocrine and enzyme markers were largely stable from baseline to 6-months after collection. Stability of acylcarnitines was variable. Greatest changes in analyte levels were observed after 12-months of storage. As a result of shifts in newborn screening analyte levels secondary to storage, the performance of metabolic gestational age algorithms was poorest when sample analysis was conducted 12-months after collection. Our models consistently demonstrated strong performance for dichotomous classification of infants as either ‘term’ or ‘preterm’, although continuous estimates of gestational age were more affected.

In North America, state and provincial policies for the retention, storage and residual use of samples collected as part of newborn screening programs vary widely, ranging from one month to decades, to indefinitely ^1^,^12^. In Ontario, Canada, samples are stored for 19 years before they are destroyed ^13^. Protein, DNA and other potential targets from dried blood spots have been shown to be stable over many years ^14^,^17^. However, the stability of individual analytes used to interpret newborn screening profiles after exposure to different storage conditions has been found to be largely variable. Available literature suggests a detrimental effect of high temperatures and high humidity on analyte concentrations. In a comprehensive study of the effect of storage conditions on 34 newborn screening markers, Adam *et al.* reported that all analytes were significantly reduced following 30-day storage at high temperature (37°C) or high humidity (>90%). The enzyme activities of GALT and BIOT were particularly susceptible, losing >60% of their initial activity when stored at high temperature, and >70% of their initial activities when stored at high humidity ^18^. Our study also confirms variability of BIOT upon retesting. A study of the stability of amino acids and acylcarnitines over 8 days also found that high temperature and humidity increased the rate of analyte degradation, but that the analyte loss was greatest within the first 24 hours of exposure ^19^.

Unique to this study is our evaluation of the impact of alterations in metabolic profiles over time on the performance of gestational age estimation models developed by our group. We have previously demonstrated the accuracy of such algorithms to estimate gestational age to within one week when applied to infants born in Ontario, Canada ^3^,^7^,^9^. Gestational age algorithms such as those described here have the potential to provide reliable population-level estimates of preterm birth for jurisdictions where such data are currently lacking ^20^. A 2017 review of the diagnostic accuracy of neonatal assessment for gestational age determination highlighted the challenges and limitations of postnatal neonatal scores which tend to overestimate gestational age in preterm infants and perform poorly in growth-restricted groups ^21^. All metabolic algorithms published to date have been developed using ultrasound gestational age as the reference standard, are not subject to user variability and have been validated in small-for-gestational-age infant subgroups. Where the goal is to identify all preterm infants, models published by our group consistently demonstrate strong performance (AUC >0.9) for distinguishing infants as ‘term’ or ‘preterm’. In contrast, continuous estimates of gestational age may be of more use on an individual level or to robustly describe a population of interest. Although continuous models published by our group demonstrate favourable performance, what constitutes ‘acceptable’ performance relative to ultrasound or LMP reference standards is yet to be determined. Recent work has focused on streamlining and tailoring published algorithms for use across a range of infant subpopulations ^7^. Validation of these models among various ethnic subgroups in Canada ^9^ and in international settings has also yielded promising results. We are currently engaged in validating published algorithms in external newborn screening cohorts from the Philippines and China.

Where there are plans to implement this technology to generate preterm birth estimates in select low- and middle-income countries ^22^, feasibility and scalability are important factors to consider. Data from this study can be used to determine the optimum length of storage of samples to manage program operations. Here, maintaining the integrity of blood spot samples prior to shipment to designated laboratories will be essential. In many parts of the world, including Sub-Saharan Africa and South East Asia, dried blood spot cards may be exposed to high temperatures and humidity during storage and transportation if immediate sample processing is unavailable. While current guidelines for newborn screening in Ontario are to analyze samples within 14 days of collection ^23^, the present study suggests that room temperature, humidity-controlled storage should be sufficient to yield reliable metabolic data for gestational age dating after 2–4 months of storage. Refrigeration of samples, if feasible, stands to extend the viable storage duration ^24^.

The strengths of this study include our use of a relatively large number of samples compared to other similarly structured studies, as well as our examination of four time-points over a wide interval of sample storage (2 to 12 months). Our use of a large number of samples of preterm infants - approximately 50% per time-point of analysis - permitted sound evaluation of gestational age estimation models. There are two notable limitations to this work. Although the study provides insight into the stability of newborn screening analytes stored in temperature and humidity-controlled conditions, we did not explore the effect of extreme environmental storage conditions on dried blood spot samples. Second, our study was limited to samples that had provided ‘negative’ screening results upon their first analysis. As it is unclear whether extremely low or high concentrations of analytes exhibit similar rates of degradation as analyte levels falling within the standard clinical reference range, as in our study, we cannot infer the stability of analyte concentrations from infants with congenital conditions.

In this paper, we have established that duration of storage, independent of temperature and humidity affect newborn screening profiles and gestational age estimates derived from metabolic gestational dating algorithms. When considering dried blood spot samples for secondary use, either for clinical or research purposes, care should be taken to store samples in temperature and humidity-controlled environments.

## Data availability

Data stemming from this project arose from a programmatic quality assurance initiative at Newborn Screening Ontario (NSO). As such, the authors do not have permissions to share the raw newborn screening data associated with this project. NSO is administered by the Children’s Hospital of Eastern Ontario (CHEO) and funded by the Ontario Ministry of Health and Long-term Care. NSO is committed to keeping newborn information, blood samples, and data arising from analysis safe and confidential. CHEO follows the following Canadian Standards Association privacy principles, which form the framework for Personal Health Information Protection Act, 2004 (PHIPA). PHIPA is Ontario's health information privacy legislation. It sets rules for how personal health information can be collected, used and disclosed. CHEO will not use or disclose personal information for purposes other than those for which it was collected, except with the consent of the individual or as required by law.

Individuals seeking a copy of the data presented in this study should contact newbornscreening@cheo.on.ca, and the request will be assessed as per NSO’s data request and secondary use policies. For more information, please visit the NSO website: https://www.newbornscreening.on.ca/en/screening-facts/screening-faq (‘What happens when a researcher wants to access stored samples for research’); https://www.newbornscreening.on.ca/en/privacy-and-confidentiality.

## Consent

NSO regularly seeks to improve existing testing. This quality assurance project sought to determine the stability of newborn samples after storage in agreement with the provincial terms of secondary use of newborn screening samples. As this was a quality improvement project, the requirement for ethics review and informed participant consent was waived by the Children’s Hospital of Eastern Ontario Research Institute.

## Supplementary material

Supplementary File 1: Pearson correlation of stored analyte profiles with baseline values; Intraclass correlation coefficients (ICC) for agreement between analyte values at baseline and after storage; P-values from Wilcoxon paired test with two-sided alternative hypothesis, on biomarkers after winsorization; Logistic regression model performance metrics.
