# Supplementary material for: Metabolic profiles derived from residual blood spot samples: A longitudinal analysis
Source: Gates Open Res. 2018 May 30;2:28. [Version 1] doi: 10.12688/gatesopenres.12822.1 (PMC6139383; doi:10.12688/gatesopenres.12822.1)
Supplement: Supplementary file 1 [file gatesopenres-2-13893-s0000.tgz › 521996a9-67ab-448e-a61f-733a435b6b41.docx]

**Supplementary File 1**

**Pearson correlation of stored analyte profiles with baseline values.**

|  | 2 Months | | | 4 Months | | | 6 Months | | | 12 Months | | |
| --- | --- | --- | --- | --- | --- | --- | --- | --- | --- | --- | --- | --- |
| **Variable** | **Corr** | **Lcl** | **Ucl** | **Corr** | **Lcl** | **Ucl** | **Corr** | **Lcl** | **Ucl** | **Corr** | **Lcl** | **Ucl** |
| HGB A | 0.995 | 0.992 | 0.997 | 0.988 | 0.980 | 0.993 | 0.990 | 0.984 | 0.994 | 0.960 | 0.924 | 0.979 |
| HGB F | 0.902 | 0.846 | 0.939 | 0.837 | 0.746 | 0.898 | 0.859 | 0.783 | 0.910 | 0.562 | 0.300 | 0.745 |
| HGB F1 | 0.860 | 0.783 | 0.911 | 0.657 | 0.492 | 0.776 | 0.792 | 0.685 | 0.865 | 0.420 | 0.121 | 0.649 |
| HGB F+F1 | 0.976 | 0.962 | 0.985 | 0.928 | 0.885 | 0.956 | 0.945 | 0.913 | 0.965 | 0.721 | 0.525 | 0.845 |
| HGB F+F1/HGB (A+F+F1) | 0.996 | 0.994 | 0.998 | 0.992 | 0.987 | 0.995 | 0.992 | 0.987 | 0.995 | 0.971 | 0.945 | 0.985 |
| 17 OHP | 0.991 | 0.986 | 0.995 | 0.984 | 0.975 | 0.990 | 0.986 | 0.978 | 0.991 | 0.972 | 0.956 | 0.982 |
| TSH | 0.974 | 0.960 | 0.984 | 0.796 | 0.695 | 0.867 | 0.777 | 0.670 | 0.852 | 0.933 | 0.897 | 0.957 |
| BIOT | 0.923 | 0.879 | 0.951 | 0.887 | 0.826 | 0.927 | 0.913 | 0.866 | 0.944 | 0.876 | 0.812 | 0.920 |
| GALT | 0.937 | 0.901 | 0.960 | 0.838 | 0.754 | 0.895 | 0.837 | 0.755 | 0.893 | 0.714 | 0.584 | 0.808 |
| TREC | 0.753 | 0.634 | 0.838 | 0.802 | 0.704 | 0.871 | 0.754 | 0.639 | 0.836 | 0.659 | 0.511 | 0.769 |
| Ala | 0.810 | 0.714 | 0.876 | 0.824 | 0.734 | 0.885 | 0.865 | 0.796 | 0.912 | 0.835 | 0.752 | 0.892 |
| Arg | 0.827 | 0.739 | 0.888 | 0.965 | 0.946 | 0.978 | 0.946 | 0.916 | 0.965 | 0.686 | 0.546 | 0.788 |
| Asa | 0.116 | -0.116 | 0.335 | 0.125 | -0.105 | 0.343 | 0.360 | 0.149 | 0.539 | 0.131 | -0.094 | 0.344 |
| Cit | 0.912 | 0.864 | 0.944 | 0.949 | 0.920 | 0.967 | 0.903 | 0.852 | 0.937 | 0.820 | 0.730 | 0.881 |
| Gly | 0.877 | 0.811 | 0.921 | 0.896 | 0.839 | 0.933 | 0.880 | 0.818 | 0.922 | 0.803 | 0.707 | 0.870 |
| Leu | 0.897 | 0.841 | 0.934 | 0.893 | 0.835 | 0.931 | 0.941 | 0.909 | 0.962 | 0.868 | 0.800 | 0.914 |
| Met | 0.807 | 0.710 | 0.874 | 0.728 | 0.600 | 0.819 | 0.846 | 0.768 | 0.899 | 0.713 | 0.583 | 0.808 |
| Orn | 0.820 | 0.727 | 0.883 | 0.772 | 0.661 | 0.850 | 0.842 | 0.763 | 0.897 | 0.631 | 0.475 | 0.748 |
| Phe | 0.880 | 0.815 | 0.923 | 0.859 | 0.784 | 0.908 | 0.913 | 0.866 | 0.944 | 0.792 | 0.692 | 0.863 |
| Tyr | 0.956 | 0.931 | 0.972 | 0.961 | 0.939 | 0.975 | 0.960 | 0.938 | 0.975 | 0.941 | 0.909 | 0.962 |
| Val | 0.876 | 0.810 | 0.920 | 0.872 | 0.805 | 0.918 | 0.916 | 0.871 | 0.946 | 0.845 | 0.767 | 0.899 |
| C0 | 0.950 | 0.922 | 0.968 | 0.948 | 0.918 | 0.967 | 0.920 | 0.877 | 0.948 | 0.900 | 0.847 | 0.935 |
| C2 | 0.960 | 0.936 | 0.974 | 0.973 | 0.958 | 0.983 | 0.941 | 0.908 | 0.962 | 0.812 | 0.720 | 0.877 |
| C3 | 0.965 | 0.944 | 0.978 | 0.970 | 0.953 | 0.981 | 0.982 | 0.971 | 0.988 | 0.853 | 0.779 | 0.904 |
| C3DC | 0.726 | 0.596 | 0.819 | 0.889 | 0.830 | 0.929 | 0.777 | 0.670 | 0.852 | 0.742 | 0.623 | 0.828 |
| C4 | 0.882 | 0.818 | 0.924 | 0.841 | 0.759 | 0.897 | 0.860 | 0.788 | 0.908 | 0.865 | 0.796 | 0.912 |
| C4DC | 0.965 | 0.945 | 0.978 | 0.971 | 0.954 | 0.982 | 0.961 | 0.939 | 0.975 | 0.871 | 0.804 | 0.916 |
| C4OH | 0.622 | 0.459 | 0.745 | 0.906 | 0.854 | 0.939 | 0.800 | 0.703 | 0.868 | 0.598 | 0.434 | 0.724 |
| C5 | 0.713 | 0.578 | 0.809 | 0.847 | 0.768 | 0.901 | 0.855 | 0.781 | 0.905 | 0.812 | 0.720 | 0.876 |
| C5:1 | 0.122 | -0.109 | 0.341 | 0.033 | -0.195 | 0.258 | -0.085 | -0.302 | 0.140 | -0.078 | -0.295 | 0.148 |
| C5DC | 0.891 | 0.832 | 0.930 | 0.927 | 0.886 | 0.953 | 0.930 | 0.893 | 0.955 | 0.857 | 0.783 | 0.906 |
| C5OH | 0.887 | 0.826 | 0.928 | 0.688 | 0.546 | 0.791 | 0.700 | 0.565 | 0.798 | 0.592 | 0.425 | 0.719 |
| C6 | 0.621 | 0.457 | 0.744 | 0.474 | 0.277 | 0.633 | 0.511 | 0.325 | 0.658 | 0.478 | 0.286 | 0.633 |
| C6DC | 0.650 | 0.494 | 0.765 | 0.857 | 0.782 | 0.907 | 0.842 | 0.762 | 0.896 | 0.695 | 0.559 | 0.795 |
| C8 | 0.615 | 0.450 | 0.739 | 0.799 | 0.698 | 0.868 | 0.640 | 0.486 | 0.755 | 0.384 | 0.177 | 0.559 |
| C8:1 | 0.674 | 0.526 | 0.782 | 0.686 | 0.543 | 0.790 | 0.834 | 0.750 | 0.891 | 0.754 | 0.639 | 0.836 |
| C10 | 0.734 | 0.607 | 0.824 | 0.778 | 0.669 | 0.854 | 0.671 | 0.527 | 0.777 | 0.669 | 0.525 | 0.776 |
| C10:1 | 0.230 | 0.002 | 0.436 | 0.334 | 0.116 | 0.522 | 0.416 | 0.213 | 0.584 | 0.252 | 0.031 | 0.449 |
| C12:1 | 0.806 | 0.708 | 0.874 | 0.888 | 0.828 | 0.928 | 0.908 | 0.859 | 0.940 | 0.892 | 0.835 | 0.930 |
| C12 | 0.809 | 0.713 | 0.876 | 0.740 | 0.616 | 0.828 | 0.807 | 0.712 | 0.873 | 0.763 | 0.651 | 0.843 |
| C14 | 0.896 | 0.839 | 0.933 | 0.810 | 0.714 | 0.876 | 0.744 | 0.625 | 0.829 | 0.739 | 0.618 | 0.826 |
| C14OH | 0.285 | 0.061 | 0.482 | 0.222 | -0.005 | 0.428 | 0.361 | 0.151 | 0.540 | 0.424 | 0.223 | 0.591 |
| C14:1 | 0.857 | 0.782 | 0.908 | 0.850 | 0.772 | 0.903 | 0.825 | 0.738 | 0.885 | 0.789 | 0.687 | 0.861 |
| C14:2 | 0.549 | 0.366 | 0.691 | 0.364 | 0.150 | 0.546 | 0.440 | 0.241 | 0.603 | 0.443 | 0.245 | 0.606 |
| C16 | 0.951 | 0.923 | 0.969 | 0.963 | 0.941 | 0.976 | 0.950 | 0.922 | 0.968 | 0.929 | 0.890 | 0.954 |
| C16OH | 0.456 | 0.254 | 0.620 | 0.370 | 0.156 | 0.551 | 0.543 | 0.365 | 0.683 | 0.280 | 0.062 | 0.473 |
| C16:1OH | 0.534 | 0.348 | 0.680 | 0.506 | 0.315 | 0.657 | 0.472 | 0.278 | 0.628 | 0.364 | 0.153 | 0.542 |
| C18 | 0.904 | 0.851 | 0.938 | 0.917 | 0.871 | 0.947 | 0.920 | 0.877 | 0.948 | 0.864 | 0.795 | 0.912 |
| C18OH | 0.419 | 0.211 | 0.591 | 0.079 | -0.151 | 0.300 | 0.140 | -0.085 | 0.351 | 0.179 | -0.045 | 0.386 |
| C18:1 | 0.930 | 0.890 | 0.955 | 0.898 | 0.842 | 0.934 | 0.936 | 0.900 | 0.959 | 0.885 | 0.826 | 0.926 |
| C18:2 | 0.883 | 0.821 | 0.925 | 0.871 | 0.802 | 0.916 | 0.907 | 0.857 | 0.940 | 0.834 | 0.751 | 0.891 |
| C18:1OH | 0.154 | -0.077 | 0.370 | 0.262 | 0.037 | 0.461 | 0.371 | 0.162 | 0.548 | 0.399 | 0.194 | 0.571 |

Pearson Correlation ≥0.8; Pearson Correlation 0.5-0.8; Pearson Correlation ≤0.5

**Intraclass correlation coefficients (ICC) for agreement between analyte values at baseline and after storage.**

|  | Baseline vs 2 months | Baseline vs 4 months | Baseline vs 6 months | Baseline vs 12 months |
| --- | --- | --- | --- | --- |
| TSH | 0.965 (0.909, 0.983) | 0.782 (0.672, 0.858) | 0.762 (0.650, 0.842) | 0.703 (-0.062, 0.901) |
| 17OHP | 0.976 (0.952, 0.987) | 0.979 (0.965, 0.987) | 0.984 (0.974, 0.99) | 0.962 (0.926, 0.979) |
| Hb ratio | 0.967 (0.107, 0.993) | 0.948 (0.112, 0.987) | 0.946 (0.121, 0.986) | 0.883 (0.006, 0.970) |
| GALT | 0.139 (-0.037, 0.435) | 0.065 (-0.042, 0.239) | 0.058 (-0.037, 0.218) | 0.025 (-0.024, 0.106) |
| BIOT | 0.443 (-0.084, 0.773) | 0.480 (-0.083, 0.799) | 0.244 (-0.053, 0.600) | 0.078 (-0.038, 0.281) |
| TREC | 0.490 (-0.035, 0.753) | 0.484 (-0.045, 0.750) | 0.278 (-0.098, 0.597) | 0.753 (0.637, 0.836) |
| Ala | 0.741 (0.419, 0.870) | 0.674 (0.116, 0.859) | 0.692 (0.048, 0.878) | 0.740 (0.317, 0.881) |
| Arg | 0.844 (0.763, 0.898) | 0.890 (0.639, 0.952) | 0.907 (0.852, 0.941) | 0.661 (0.515, 0.770) |
| Asa | 0.104 (-0.118, 0.320) | 0.089 (-0.081, 0.270) | 0.195 (-0.069, 0.436) | 0.043 (-0.055, 0.167) |
| Cit | 0.850 (0.425, 0.941) | 0.855 (0.093, 0.955) | 0.875 (0.732, 0.934) | 0.552 (-0.084, 0.819) |
| Gly | 0.840 (0.654, 0.916) | 0.741 (0.044, 0.906) | 0.785 (0.262, 0.913) | 0.455 (-0.092, 0.776) |
| Leu | 0.845 (0.546, 0.931) | 0.830 (0.529, 0.922) | 0.903 (0.604, 0.962) | 0.755 (0.181, 0.902) |
| Met | 0.324 (-0.072, 0.681) | 0.418 (-0.096, 0.723) | 0.649 (-0.046, 0.868) | 0.182 (-0.065, 0.496) |
| Orn | 0.530 (-0.089, 0.807) | 0.303 (-0.099, 0.618) | 0.366 (-0.100, 0.681) | 0.106 (-0.063, 0.336) |
| Phe | 0.721 (0.072, 0.893) | 0.510 (-0.090, 0.812) | 0.874 (0.638, 0.942) | 0.386 (-0.082, 0.731) |
| Suac | 0.515 (0.325, 0.665) | 0.084 (-0.041, 0.297) | 0.276 (-0.077, 0.553) | 0.059 (-0.033, 0.225) |
| Tyr | 0.900 (0.393, 0.966) | 0.828 (-0.013, 0.950) | 0.948 (0.904, 0.970) | 0.791 (0.072, 0.929) |
| Val | 0.852 (0.727, 0.916) | 0.796 (0.549, 0.895) | 0.857 (0.465, 0.942) | 0.805 (0.608, 0.894) |
| C0 | 0.934 (0.834, 0.967) | 0.927 (0.810, 0.965) | 0.887 (0.707, 0.946) | 0.714 (-0.054, 0.905) |
| C2 | 0.547 (-0.060, 0.847) | 0.760 (-0.050, 0.936) | 0.471 (-0.066, 0.801) | 0.043 (-0.039, 0.166) |
| C3 | 0.838 (0.020, 0.952) | 0.962 (0.919, 0.980) | 0.891 (0.062, 0.970) | 0.138 (-0.067, 0.408) |
| C3DC | 0.617 (0.225, 0.799) | 0.764 (0.081, 0.915) | 0.289 (-0.082, 0.635) | 0.150 (-0.070, 0.430) |
| C4 | 0.836 (0.600, 0.919) | 0.827 (0.738, 0.888) | 0.855 (0.782, 0.905) | 0.654 (0.050, 0.855) |
| C4DC | 0.965 (0.945, 0.978) | 0.668 (-0.078, 0.891) | 0.932 (0.805, 0.968) | 0.525 (-0.094, 0.815) |
| C4OH | 0.374 (-0.080, 0.666) | 0.584 (-0.078, 0.838) | 0.483 (-0.096, 0.780) | 0.074 (-0.058, 0.248) |
| C5 | 0.706 (0.570, 0.804) | 0.839 (0.757, 0.895) | 0.852 (0.777, 0.903) | 0.807 (0.713, 0.872) |
| C5:1 | 0.126 (-0.104, 0.344) | 0.182 (-0.045, 0.391) | -0.087 (-0.298, 0.134) | -0.004 (-0.220, 0.214) |
| C5DC | 0.840 (0.576, 0.925) | 0.910 (0.824, 0.950) | 0.900 (0.722, 0.954) | 0.456 (-0.089, 0.779) |
| C5OH | 0.793 (0.621, 0.882) | 0.589 (0.290, 0.759) | 0.700 (0.567, 0.798) | 0.288 (-0.096, 0.591) |
| C6 | 0.605 (0.435, 0.733) | 0.462 (0.266, 0.621) | 0.511 (0.327, 0.658) | 0.401 (0.132, 0.601) |
| C6DC | 0.548 (0.257, 0.725) | 0.486 (-0.096, 0.782) | 0.372 (-0.099, 0.700) | 0.218 (-0.092, 0.502) |
| C8 | 0.600 (0.432, 0.728) | 0.773 (0.664, 0.850) | 0.614 (0.447, 0.738) | 0.310 (0.067, 0.511) |
| C8:1 | 0.640 (0.454, 0.767) | 0.602 (0.282, 0.773) | 0.827 (0.741, 0.886) | 0.617 (0.224, 0.799) |
| C10 | 0.664 (0.428, 0.800) | 0.756 (0.637, 0.839) | 0.582 (0.286, 0.752) | 0.520 (0.147, 0.727) |
| C10:1 | 0.228 (0.007, 0.430) | 0.288 (0.071, 0.481) | 0.378 (0.163, 0.557) | 0.300 (0.089, 0.486) |
| C12 | 0.746 (0.465, 0.867) | 0.700 (0.531, 0.809) | 0.715 (0.413, 0.849) | 0.711 (0.526, 0.822) |
| C12:1 | 0.793 (0.683, 0.866) | 0.879 (0.814, 0.922) | 0.905 (0.855, 0.939) | 0.782 (0.517, 0.888) |
| C14 | 0.867 (0.743, 0.926) | 0.766 (0.586, 0.863) | 0.664 (0.357, 0.815) | 0.583 (0.125, 0.790) |
| C14OH | 0.280 (0.061, 0.474) | 0.201 (-0.007, 0.398) | 0.329 (0.115, 0.514) | 0.395 (0.189, 0.567) |
| C14:1 | 0.838 (0.738, 0.900) | 0.837 (0.746, 0.896) | 0.705 (0.267, 0.861) | 0.647 (0.284, 0.813) |
| C14:2 | 0.529 (0.342, 0.676) | 0.301 (0.084, 0.492) | 0.442 (0.243, 0.605) | 0.402 (0.193, 0.574) |
| C16 | 0.933 (0.854, 0.965) | 0.962 (0.941, 0.976) | 0.946 (0.915, 0.966) | 0.911 (0.827, 0.951) |
| C16:1OH | 0.531 (0.347, 0.676) | 0.420 (0.076, 0.646) | 0.434 (0.238, 0.596) | 0.361 (0.151, 0.540) |
| C16:OH | 0.452 (0.249, 0.616) | 0.346 (0.133, 0.529) | 0.514 (0.332, 0.660) | 0.288 (0.070, 0.480) |
| C18 | 0.899 (0.842, 0.935) | 0.916 (0.870, 0.946) | 0.919 (0.877, 0.948) | 0.727 (0.260, 0.878) |
| C18:1 | 0.917 (0.857, 0.951) | 0.894 (0.838, 0.932) | 0.936 (0.901, 0.958) | 0.846 (0.683, 0.917) |
| C18:1OH | 0.121 (-0.112, 0.341) | 0.232 (0.015, 0.431) | 0.385 (0.178, 0.559) | 0.373 (0.164, 0.550) |
| C18:2 | 0.882 (0.819, 0.924) | 0.867 (0.797, 0.914) | 0.905 (0.856, 0.939) | 0.730 (0.530, 0.840) |
| C18:OH | 0.418 (0.214, 0.587) | 0.084 (-0.084, 0.264) | 0.065 (-0.160, 0.282) | 0.148 (-0.064, 0.351) |

ICC run assuming subjects and raters randomly were chosen from a bigger pool of persons ('"twoway"' model), with 95% confidence intervals

**P-values from Wilcoxon paired test with two-sided alternative hypothesis, on biomarkers after winsorization**

|  | Baseline vs 2 months | Baseline vs 4 months | Baseline vs 6 months | Baseline vs 12 months |
| --- | --- | --- | --- | --- |
| TSH | 5.215e-06 | 0.002784 | 0.002714 | 1.959e-13 |
| 17OHP | 2.58e-06 | 0.005611 | 0.2624 | 4.553e-05 |
| Hb ratio | 5.331e-13 | 4.298e-12 | 3.962e-13 | 7.276e-12 |
| GALT | 7.892e-14 | 5.388e-14 | 1.715e-14 | 1.715e-14 |
| BIOT | 8.565e-14 | 5.61e-14 | 1.715e-14 | 1.715e-14 |
| TREC | 2.596e-12 | 9.089e-14 | 1.927e-14 | 0.1275 |
| Ala | 1.983e-08 | 5.733e-11 | 1.364e-12 | 2.964e-10 |
| Arg | 0.0367 | 5.759e-09 | 0.03054 | 0.414 |
| Asa | 0.05478 | 7.008e-07 | 3.202e-11 | 4.576e-14 |
| Cit | 1.602e-09 | 2.128e-12 | 9.776e-07 | 2.253e-12 |
| Gly | 1.054e-07 | 2.014e-13 | 5.766e-11 | 1.715e-14 |
| Leu | 4.744e-09 | 8.706e-08 | 2.815e-09 | 3.857e-11 |
| Met | 7.893e-14 | 6.504e-13 | 2.273e-13 | 1.715e-14 |
| Orn | 2.404e-12 | 5.61e-14 | 1.715e-14 | 1.715e-14 |
| Phe | 2.867e-11 | 7.144e-14 | 2.72e-08 | 1.853e-14 |
| Suac | 0.8294 | 5.378e-14 | 7.198e-12 | 1.713e-14 |
| Tyr | 3.982e-11 | 7.437e-14 | 0.0002514 | 2.033e-13 |
| Val | 8.214e-05 | 1.694e-06 | 8.871e-10 | 3.491e-06 |
| C0 | 1.056e-06 | 3.243e-06 | 6.334e-08 | 4.325e-14 |
| C2 | 7.893e-14 | 5.388e-14 | 1.715e-14 | 1.715e-14 |
| C3 | 7.887e-14 | 9.453e-07 | 1.713e-14 | 1.713e-14 |
| C3DC | 1.444e-07 | 2.572e-10 | 3.435e-14 | 2.285e-14 |
| C4 | 6.126e-07 | 0.06538 | 0.7742 | 4.191e-12 |
| C4DC | 0.2999 | 5.314e-14 | 1.983e-07 | 1.805e-14 |
| C4OH | 1.669e-12 | 1.196e-13 | 9.191e-14 | 1.701e-14 |
| C5 | 0.05996 | 0.8766 | 0.1015 | 0.1606 |
| C5:1 | 0.2931 | 0.2842 | 0.1524 | 0.02069 |
| C5DC | 2.544e-07 | 0.0008266 | 7.995e-07 | 3.664e-14 |
| C5OH | 0.0001203 | 2.609e-06 | 0.404 | 1.408e-12 |
| C6 | 0.02718 | 0.04462 | 0.2678 | 1.811e-07 |
| C6DC | 5.081e-06 | 1.526e-12 | 4.275e-13 | 1.552e-13 |
| C8 | 0.6032 | 0.5887 | 0.00627 | 4.991e-07 |
| C8:1 | 0.001587 | 3.461e-07 | 0.636 | 1.52e-08 |
| C10 | 1.813e-05 | 0.02138 | 8.354e-07 | 2.414e-09 |
| C10:1 | 0.09151 | 0.0002639 | 0.0004264 | 0.03594 |
| C12 | 3.449e-06 | 0.0008543 | 4.715e-07 | 0.0001102 |
| C12:1 | 0.008054 | 0.1372 | 0.04609 | 7.738e-07 |
| C14 | 0.0005333 | 7.21e-05 | 9.606e-07 | 3.334e-09 |
| C14OH | 0.03316 | 0.001111 | 0.3242 | 0.00344 |
| C14:1 | 0.001855 | 0.01282 | 7.102e-09 | 6.867e-08 |
| C14:2 | 0.01381 | 0.0006175 | 0.7822 | 0.001858 |
| C16 | 3.97e-06 | 0.5349 | 0.009872 | 8.237e-05 |
| C16:1OH | 0.2656 | 9.836e-08 | 0.0698 | 0.5993 |
| C16:OH | 0.9777 | 0.1798 | 0.03823 | 0.5473 |
| C18 | 0.03639 | 0.2138 | 0.1271 | 1.417e-10 |
| C18:1 | 0.0002945 | 0.2636 | 0.2126 | 1.213e-05 |
| C18:1OH | 0.9277 | 0.0491 | 0.7498 | 0.8450 |
| C18:2 | 0.2058 | 0.2492 | 0.1927 | 1.492e-05 |
| C18:OH | 0.09182 | 1.302e-06 | 0.1609 | 0.01386 |

**Logistic regression model performance metrics**

|  | **Baseline** | **2 Month Storage** | **4 Month Storage** | **6 Month Storage** | **12 Month Storage** |
| --- | --- | --- | --- | --- | --- |
|  | **n=275** | **n=60** | **n=61** | **n=65** | **n=38** |
| **Model 1:** Clinical Model | 0.928 (0.893, 0.962) | 0.871  (0.750, 0.993) | 0.917 (0.834, 0.999) | 0.993  (0.972, 1.000) | 0.949 (0.866, 1.000) |
| **Model 2:** Metabolite Model | 0.968 (0.945, 0.991) | 0.970 (0.909, 1.000) | 0.981 (0.940, 1.000) | 0.995 (0.977, 1.000) | 0.955 (0.876, 1.000) |

Data are presented as area under the receiver operator curve (95% confidence limits)
