# Supplementary material for: Metabolic profiles derived from residual blood spot samples: A longitudinal analysis
Source: Gates Open Res. 2018 May 30;2:28. [Version 1] doi: 10.12688/gatesopenres.12822.1 (PMC6139383; doi:10.12688/gatesopenres.12822.1)
Supplement: Peer review history

# Peer review: Referee response for version 1

**DOI:** 10.21956/gatesopenres.13893.r26615

**Published:** 2018-08-28

**Authors:** Mohd M. Khan

**Affiliations:** 1 University of Maryland, Baltimore, MD, USA

The manuscript "Metabolic profiles derived from residual blood spot samples: A longitudinal analysis" by Murphy *et al *evaluates how storage conditions on archival samples correlate with reliable metabolic gestational age estimation. The study employs a relatively large cohort (n=307) with samples analyzed, post-storage for 2-, 4-, 6-, and 12-months. Authors analyzed hemoglobin profiles as well as numerous newborn analytes' profiles and metabolic gestational age algorithms were used to evaluate their performance in estimating gestational age. The greatest levels of changes due to storage conditions, were found in samples that were stored for 12 months.

 The manuscript is well written, however I have a few suggestions: 

- The statement "Duration of sample storage, independent of temperature and humidity, affects newborn screening profiles and gestational age estimates derived from metabolic gestational dating algorithms" can be revised as in the present work authors only looked at one storage condition (21°C "in temperature and humidity-controlled conditions"). I also suggest, authors include details about storage humidity levels.
- Method details for "amino acid and acylcarnitine analysis was performed by electrospray ionization tandem mass spectrometry" can be expanded and described in detail, including details of separation conditions and MS data acquisition parameters.
- Details about how to get access of "metabolic gestational dating algorithms" can be added.

In conclusion, the present work successfully evaluatse the impact of storage time (up to 12-months) and future longitudinal studies can look at >12 months storage and/or different storage conditions.

I have read this submission. I believe that I have an appropriate level of expertise to confirm that it is of an acceptable scientific standard.

# Peer review: Referee response for version 1

**DOI:** 10.21956/gatesopenres.13893.r26491

**Published:** 2018-06-18

**Authors:** Pratik Panchal

**Affiliations:** 1 OpenBiome, Somerville, MA, USA

I think the authors do a great job at validating the long-term data obtained from blood spot samples. Further, the characterization of the variability over the 4 time points is a useful tool for clinicians in low resource settings. 

 Comparing the Model 1 vs 2 further highlights the poor quality of gestational age information that can be derived from models that only focus on clinical variables. 

 With respect to future direction, this project must be scaled up to include data on outliers (which would represent patients with congenital conditions) and characterize the variability of the analytes at the same four time points mentioned in this paper. 

 Overall, the authors have taken the challenge of better predicting GA using clinical and analyte variables and successfuly demontrate the performance of their model vs the classical clinical model.

I have read this submission. I believe that I have an appropriate level of expertise to confirm that it is of an acceptable scientific standard.
